# Measuring differentiation among populations at different levels of genetic integration

**DOI:** 10.1186/1471-2156-9-60

**Published:** 2008-09-30

**Authors:** Elizabeth M Gillet, Hans-Rolf Gregorius

**Affiliations:** 1 Abteilung Forstgenetik und Forstpflanzenzüchtung, Universität Göttingen, Büsgenweg 2, 37077 Göttingen, Germany; 2 Institut für Populations- und ökologische Genetik (IPOEG), Am Pfingstanger 58, 37075 Göttingen, Germany

## Abstract

**Background:**

Most genetic studies of population differentiation are based on gene-pool frequencies. Population differences for gene associations that show up as deviations from Hardy-Weinberg proportions (homologous association) or gametic disequilibria (non-homologous association) are disregarded. Thus little is known about patterns of population differentiation at higher levels of genetic integration nor the causal forces.

**Results:**

To fill this gap, a conceptual approach to the description and analysis of patterns of genetic differentiation at arbitrary levels of genetic integration (single or multiple loci, varying degrees of ploidy) is introduced. Measurement of differentiation is based on the measure Δ of genetic distance between populations, which is in turn based on an elementary genic difference between individuals at any given level of genetic integration. It is proven that Δ does not decrease when the level of genetic integration is increased, with equality if the gene associations at the higher level follow the same function in both populations (e.g. equal inbreeding coefficients, no association between loci). The pattern of differentiation is described using the matrix of pairwise genetic distances Δ and the differentiation snail based on the symmetric population differentiation Δ_*SD*_. A measure of covariation compares patterns between levels. To show the significance of the observed differentiation among possible gene associations, a special permutation analysis is proposed. Applying this approach to published genetic data on oak, the differentiation is found to increase considerably from lower to higher levels of integration, revealing variation in the forms of gene association among populations.

**Conclusion:**

This new approach to the analysis of genetic differentiation among populations demonstrates that the consideration of gene associations within populations adds a new quality to studies on population differentiation that is overlooked when viewing only gene-pools.

## Background

Most biological species are subdivided into populations that are more or less strongly connected by gene flow. This facilitates a species' persistence via adaptive differentiation to local conditions, which in turn serves to maintain genetic variation for future adaptational processes. This concept of species is reflected, for example, in meta-population analysis with its special emphasis on extinction-recolonization dynamics (see [1] for a still relevant review). Genetic control of the phenotypic traits on which processes of adaptation operate is usually complex due to the involvement of several interacting genetic traits that may be expressed even in different developmental phases, including the haplophase. The detection of selectively neutral impacts on population differentiation (e.g. founder effects, genetic drift) may also require the analysis of multiple genetic traits, the interactions among which are determined by chance and in combination with particular mating systems (such as partial selfing). Thus the amount and pattern of genetic differentiation among a set of populations basically depends on:

(1) the developmental stage (chiefly haplophase *vs*. diplophase),

(2) the genetic traits under consideration at this stage, and

(3) the ways in which the different states of these genetic traits in the populations are associated to form the genetic types (haplotypes, genotypes) at this stage, broadly termed *gene association *in this paper.

In general, traits are genetic only if they are inheritable, and the goal of inheritance analysis is to identify genes as the basic units of inheritance. The term *genetic integration *is used here to designate the combination or arrangement of these elementary objects "gene" into the haplotypes of gametes, into the genotypes at diploid (or polyploid) nuclei of diplophase individuals, or into the cytotypes of mitochondria or plastids, for example. Accordingly, each level of genetic integration usually corresponds to a developmental stage or an organelle that is characterized by special combinations of genes. (To emphasize this aspect, *genic integration *might be the more appropriate term.)

The main motivation for this paper was the realization that impacts of particular forces, selective or not, on population differentiation may not be observable at every level of genetic integration. Measurements of differentiation among populations based on gene frequencies, for example, provide no specific insights into the effects of mating systems nor of epistatic interaction on population differentiation. This is due to the fact that gene frequencies refer to the lowest level of genetic integration, namely its absence. This level, which is commonly addressed as a population's gene-pool, is conceived to consist of the set of all individual genes present in the population members for a specified set of genetic traits. Genetic studies of population differentiation are almost always based on this "beanbag" (critically reflected by Mayr [2] and defended by Haldane [3]; for concise reasoning of the persistence of the gene-pool concept see e.g. [4] or [5]). Studies of differentiation at multiple loci are no exception, since they commonly report averages over single-locus differentiation indices. Also disregarded in studies of gene-pool differentiation are gene associations that deviate from Hardy-Weinberg proportions (homologous, or intralocus, association) or gametic equilibria (non-homologous, or interlocus, association). Considering that forms and degrees of gene association may differ at different levels of genetic integration, it thus appears that previous studies on patterns of population differentiation have provided very little information on levels of genetic integration above the gene-pool.

One important reason for the usual focus on gene-pool differentiation is probably the lack of a method for measuring population differentiation consistently at all levels of genetic integration. Consistency means that comparison of the amount of differentiation among a set of populations between levels of integration provides information about the complexity of the gene associations that distinguish them. Since gene associations do not decrease as level of integration increases, neither should differentiation. Moreover, the extent of an increase in differentiation between subsequent levels should in some way reflect the degree of complexity of the additional gene associations, with equality as an indication of lack of additional complexity by some standard. Such a differentiation measure must thus be based on a conceptual characterization of the complexity of gene associations.

The existence of such a measure would not only facilitate experimental studies but also simplify the development and testing of models. Insights can be gained from models only when the characteristics described by the models derive from concepts that are conceived independently of the models. Thus models do not serve to analyze characteristics: characteristics serve to analyze models. Moreover, model-based analysis that is limited to falsification of a particular model or its parameterization provides no information on the validity of related models. A conceptually argued measure, in contrast, can be applied to whole classes of models. This permits summarization of characteristics they have in common, the statistical significance of which can be tested by permutation analysis.

In the present paper, a new approach to differentiation analysis is presented that applies a conceptually argued measure of differentiation Δ_*SD *_to analyze and compare differentiation patterns among populations at different levels of integration. Presentation includes the development of Δ_*SD*_, representation of patterns of differentiation, and tests of significance of the patterns. Comparison of differentiation between levels of integration is analyzed mathematically. The method's usefulness is demonstrated by applying it to six-locus microsatellite data from four stands of pedunculate oak (*Quercus robur*). The purpose of using real data is to show how insights can be gained directly from observations without limitation to particular models, the testability of which may be difficult. It turned out that the large increases in differentiation between levels that were observed in the real data were not producible in numerous simulations of simple selection models, indicating that these models cannot explain the complexity of the real data. Studies of the behavior of this measure using simulated data from increasingly complex models will be the subject of a future paper.

To prevent possible misunderstanding, it should be mentioned that this approach differs in content from any type of (hierarchical) partitioning, apportionment, or allocation of genetic variation (such as within and between populations). Methods of attributing overall variation to partitions draw upon the principle of the analysis of variance and were extended to include more general measures of difference between individuals by Rao (equation 2.3.1 in [6]). An application of this generalization to a special measure of genetic difference for multiple loci between haplotypes led Excoffier *et al*. [7] to the formulation of their "analysis of molecular variance". In contrast, the levels of genetic integration dealt with here cannot serve as classes (partitions) over which genetic variation is distributed. Instead, at each integration level (e.g. gene pool, single-locus genotypes, multilocus genotypes) the genetic characteristics can be analyzed for their differentiation within population subdivisions. Subsequent comparison between levels reveals which level of integration, and thus which type of gene association (especially homologous *vs*. non-homologous), has the greatest influence on the differentiation within the partition.

## Methods

### Levels of genetic integration and gene association

At the lowest level of genetic integration, the gene-pool, the *gene-type *of each individual gene is characterized by the gene locus at which it is located and by its allelic state. Assuming that the degree of ploidy is the same at all loci, the relative frequencies of the gene-types in the gene-pool of a population equal $\frac{1}{L}$·*p*_*i*;*l*_, where *L *is the number of loci and *p*_*i*;*l *_is the relative frequency of the *i*-th allele at the *l*-th gene locus in the population (∑_*i*_*p*_*i*;*l *_= 1, $\sum_{i;l}\frac{1}{L}\cdot p_{i;l}=1$). If loci of differing degree of ploidy (e.g. nuclear and organelle) are included in the analysis, replace $\frac{1}{L}$ with the locus-specific quantities *r*_*l *_obtained by division of the degree of ploidy at the *l*-th locus by the sum of the degrees over all loci. The gene-pool frequency of the gene-type specified by the *i*-th allele at the *l*-th locus then equals *r*_*l*_·*p*_*i*;*l*_. At higher levels of genetic integration, where the objects of interest represent compositions of several individual genes together with their gene-types, association among gene-types becomes relevant for differentiation studies. If the objects are diplophase individuals and if the gene-types are specified at a single gene-locus, then all associations among the genes that make up the genotypes are homologous (*i.e*., allelic) by definition. When multiple loci are considered, both homologous and non-homologous (interlocus) associations exist among genes. If the objects are haplophases, each object having just one gene per locus, then all gene associations are non-homologous. Since at any given locus all objects carry the number of (allelic) individual genes specified by the degree of ploidy of the locus, the objects representing a given level of genetic integration are characterized by the same number of individual genes.

### The elementary genic difference

From this perspective, genetic differences between two objects of the same level of integration are basically determined by the number of their individual genes that differ in type. If the numbers of copies of the *i*-th allele at the *l*-th gene locus are denoted by *n*_*i*;*l *_and *m*_*i*;*l*_, respectively, then the two objects differ by ∑_*i*,*l*_|*n*_*i*;*l *_- *m*_*i*;*l*_| gene-type copies. This sum is maximal, equaling two times the total number *K *of individual genes represented in each object, if the objects share no gene-types (and thus differ completely). Since ∑_*i*,*l*_*n*_*i*;*l *_= ∑_*i*,*l*_*m*_*i*;*l *_= *K *holds, division of ∑_*i*,*l *_|*n*_*i*;*l *_- *m*_*i*;*l*_| by 2·*K *yields a measure of genic difference that is bounded between zero and one. This measure of *elementary genic difference *is applicable to all levels of integration. It differs from a closely related index suggested by Smouse and Peakall [8] in a different context, in which the absolute difference is replaced by the squared difference, a disadvantage of which is that objects sharing no gene-types need not realize the maximum difference.

The elementary genic difference does not distinguish homologous from non-homologous genes. Hence, the homologous and non-homologous gene arrangements within the objects affect the elementary genic differences between them only through their sum. For example, in the case of diploid individuals scored at two gene loci *A *and *B*, say, the genotypes *A*_1_*A*_1_/*B*_1_*B*_2 _and *A*_1_*A*_2_/*B*_1_*B*_3 _represent three (*A*_1_, *B*_1_, *B*_2_) and four (*A*_1_, *A*_2_, *B*_1_, *B*_3_), respectively, of the total of five gene-types. *A*_1 _is represented by two copies in the first genotype and by one copy in the second, and the remaining four gene-types are represented by at most one copy in each of the two genotypes. The sum of copy number differences between the two genotypes thus equals four. After division by twice the number of individual genes in a genotype (i.e. 2·4), this yields 0.5 as the elementary genic difference. The same result is obtained for the two genotypes *A*_1_*A*_2_/*B*_1_*B*_2 _and *A*_1_*A*_2_/*B*_3_*B*_3_, even though all genic differences are now due to the alleles at a single locus (*B*).

These considerations show that objects representing higher levels of genetic integration are not simply of the same or different genetic type, as is the case at the level of the gene-pool. Specification of the gene-types of which the genetic types are composed yields a measure of the differences between them that ensures the comparability of genetic differences even across levels of genetic integration. Thus, analysis of population differentiation at higher levels of integration should take into account not only differences in the frequencies of the genetic types among populations but also the variation in the pairwise differences between types.

### The measure Δ of genetic distance between two populations

The measure Δ of genetic distance between two populations developed by Gregorius *et al*. [9] considers both the frequencies of genetic types and their pairwise differences, while avoiding the conceptual problems of dispersion indices (e.g. average differences within and between populations, see [6]). For a specified trait, Δ equals the minimum extent to which the genetic types of individuals in one of the two populations must be altered in order to obtain the composition of genetic types in the other. Denote:

$$\Delta (s)=\sum_{a,b}s(a,b)\cdot d(a,b)$$

where *d*(*a*, *b*) specifies the difference between genetic types *a *and *b*, and *s*(*a*, *b*) is a frequency shift. Frequency shifts are performed from types that are more frequent in the one population P than in the other Q to types that are less frequent in P than in Q. If the frequency *p*_*a *_of type *a *in P exceeds the frequency *q*_*a *_of this type in Q, then the excess *p*_*a *_- *q*_*a *_must be shifted to types deficient in P, such that ∑_*b*_*s*(*a*, *b*) = *p*_*a *_- *q*_*a *_= *p*_*a *_- min{*p*_*a*_, *q*_*a*_}. The shift process is continued for all types with a frequency excess in P until the frequencies of all types in P match those in Q. Since there may be many different ways of shifting, Δ is taken to be the minimum of the above sum over all admissible frequency shifts *s*, *i.e*.,

$$\Delta =\underset{s}{\min}\Delta (s)$$

In [9] and [10] it is shown that finding a shift transformation *s *that minimizes Δ(*s*) is equivalent to solving the "Transportation Problem" [11] by linear programming methods. These methods are implemented in the computer program *DeltaS *[12].

In combination with the measure of elementary genic difference, the measure Δ provides the desired conceptual method for studying population differentiation at different levels of genetic integration. At the lowest integration level, the gene-pool, where gene-types are specified by indices *i*; *l *and their frequencies in populations P and Q as *r*_*l*_·*p*_*i*;*l *_and *r*_*l*_·*q*_*i*;*l *_(see above), Δ assumes a familiar form. Since individual genes are distinguished only by their identity or non-identity in type, one obtains elementary genic differences *d*(*a*, *b*) = 1 for *a *≠ *b *and *d*(*a*, *b*) = 0 for *a *= *b*. For any frequency shift *s*, it holds that Δ(*s*) = ∑_*a*, *b*_*s*(*a*, *b*) = ∑_*a*_(*p*_*a *_- min{*p*_*a*_, *q*_*a*_}) = $\frac{1}{2}$ ∑_*a*_|*p*_*a *_- *q*_*a*_|. Insertion of the gene-type notation in place of the *a*'s then yields:

$$\Delta =\sum_{l}r_{l}\cdot d_{0}(p^{\left(l\right)},q^{\left(l\right)})$$

where:

$$d_{0}(p^{\left(l\right)},q^{\left(l\right)})=\frac{1}{2}\sum_{i}\left|p_{i;l}-q_{i;l}\right|$$

In this expression, *d*_0_(***p***^(*l*)^, ***q***^(*l*)^) is a familiar measure of genetic distance between two populations with allele frequencies ***p***^(*l*)^and ***q***^(*l*) ^at locus *l *(see e.g. [13]). It turns out that the gene-pool distance between two populations equals the average distance over the single loci.

At the diplophase level of integration, for example, consider two populations P and Q with Hardy-Weinberg proportions (HWP) for the two alleles *A*_1 _and *A*_2 _at a locus. Let *p*_1 _> *q*_1_, and let P have more heterozygotes than Q. Then there is only one way *s *of shifting, namely *s*(*A*_1_*A*_1_, *A*_2_*A*_2_) = $p_{1}^{2}-q_{1}^{2}$ > 0 and *s*(*A*_1_*A*_2_, *A*_2_*A*_2_) = 2*p*_1_*p*_2 _- 2*q*_1_*q*_2 _> 0. Since for the elementary genic distance, *d*(*A*_1_*A*_1_, *A*_2_*A*_2_) = 1.0 and *d*(*A*_1_*A*_2_, *A*_2_*A*_2_) = 0.5, the genetic distance equals Δ = 1.0·($p_{1}^{2}-q_{1}^{2}$) + 0.5·(2*p*_1_*p*_2 _- 2*q*_1_*q*_2_) = *p*_1 _- *q*_1_. In this example, the distance at the diplophase level equals the gene-pool distance. Under Results it is shown (Proposition 1) that the diplophase distance is never less than the gene-pool distance and that equality at the two levels is of particular interest.

### Patterns of differentiation among populations

At this point, each level of integration for a set of populations is characterized by a matrix of pairwise distances Δ between the populations. These matrices and the relationships among them can be called the *pattern of differentiation *among the populations. Three approaches to the description of differentiation patterns are discussed.

#### Clustering methods

Matrices of pairwise genetic distances between populations are commonly represented using *clustering methods *as dendrograms, the topologies (cluster structures) of which are of primary interest. In particular, the emergence of new cluster structures at higher levels of integration emphasizes the necessity to consider evolutionary forces of population differentiation that go beyond those conventionally held responsible for gene-pool differentiation. Detection of such structures of course depends on comparison of the dendrograms from different levels of integration, where the gene-pool constitutes the basic reference for comparison. There are many ways of comparing dendrograms obtained with the same clustering method (for an overview see e.g. [14], p. 94ff). We will concentrate instead on direct comparison of the quantities underlying all methods of clustering, *i.e*., the matrix of pairwise distances. Changes in topology are most likely to occur when the distance matrices show poor correspondence across levels of integration, that is, low covariation (see below).

#### Variance decomposition

Another common approach is less detailed and essentially rests on the computation of a single statistic of the degree of differentiation among populations. Among these measures, most of which are indexed by _*ST*_, the classical versions *F*_*ST *_[15] and *G*_*ST *_[16] consider population differentiation solely for allele frequencies. More recent versions such as Φ_*ST *_[7] or *R*_*ST *_[17] include variable differences between genetic types. Inferences on patterns of differentiation are more or less restricted to ways in which an observed amount of differentiation could have evolved under certain model assumptions. Moreover, the whole family of _*ST*_-measures is based on the principle of ***variance decomposition***, where the difference between the total variation and the average variation within populations is divided by the total variation. Such measures do not assume their maximum values only for completely differentiated populations. This follows directly from their conceptual underpinning, which refers to partitioning rather than differentiation of genetic variation among populations. The _*ST*_-measures therefore have limited relevance as indicators of patterns of differentiation among populations.

#### Symmetric population differentiation Δ_*SD*_

For this reason, preference is given here to a related but more detailed approach that refers to the concept of *symmetric set difference *[18,19]. In this concept, each population is characterized by its genetic distance from its complement, *i.e*., the totality (union) of the remaining populations. By this means, populations can be ranked according to their contributions to the overall amount of differentiation. Application of the distance measure Δ to the concept of symmetric set difference yields quantities Δ_*j *_as the distance Δ(***p***(*j*), $\bar{p}$(*j*)) between the *j*-th population P(*j*) and its complement $\bar{P}$(*j*). Denoting ***p***(*j*) as the vector of type frequencies characterizing the *j*-th population, the vector $\bar{p}$(*j*) of type frequencies that represent the remaining populations equals ∑_*k*:*k*≠*j *_***p***(*j*)·*c*(*k*)/$\bar{c}$(*j*), where *c*(*k*) is the relative size of the *k*-th population and $\bar{c}$(*j*) = ∑_*k*:*k*≠*j *_*c*(*k*). With these quantities, the measure of *symmetric population differentiation *Δ_*SD *_results as the average of the single-population differentiations Δ_*j*_, *i.e*.,

$$\Delta_{SD}=\sum_{j}c(j)\cdot \Delta_{j}$$

Whereas Δ_*SD *_quantifies the average degree to which individual populations differ from their complements, its components Δ_*j *_identify individual populations as being more or less representative of the whole collection of populations. Thus, Δ_*j *_= 0 summarizes the situation where the *j*-th population perfectly represents the totality of the populations. On the other hand, the more distinctly Δ_*j *_exceeds Δ_*SD*_, the more a population is distinguished from all the others. The extreme of complete differentiation of course requires a definite notion of complete difference between types (as is the case with binary difference measures as well as with the measure *d *of elementary genic difference).

The differentiation pattern inherent in Δ_*SD *_and its components Δ_*j *_for variable population sizes can be illustrated as a "*differentiation snail*" [18] (see Fig. 2 below). The snail complements the pattern characteristics obtainable from clustering methods or directly from the distance matrix in that it reveals tendencies of population assemblages to be genetically dispersed or to concentrate genetic variation in a few populations. In order to assess changes in the snail between levels of genetic integration, the following measure of covariation of the respective components Δ_*j *_can be applied.

### Covariation of differentiation between integration levels

The degree of correspondence between differentiation indices from two levels of integration can be determined by a measure of ***covariation***. Commonly chosen measures of covariation are any of the versions of the product-moment correlation which are designed to quantify the closeness to a linear type of covariation between two variables. However, since our genetic distances are bounded, linear relationships can be realized only under very exceptional conditions. Moreover, it is difficult to see how relationships between levels of integration could be brought about by forces acting linearly on genetic distances. From this perspective it is preferable to use a measure of covariation that relies on general monotonic relationships between two variables. Such measures would more reliably detect any consistency of patterns of differentiation over levels of integration. As was pointed out in [20], a suitable measure of covariation is:

$$C=\frac{\sum_{i<k}\left(X_{i}-X_{k})\cdot (Y_{i}-Y_{k}\right)}{\sum_{i<k}\left|(X_{i}-X_{k})\cdot (Y_{i}-Y_{k})\right|}$$

where the variables *X*_*i *_and *Y*_*i *_refer to genetic distances at two different levels of integration. In the case of the distances between a population and its complement, *X*_*i *_and *Y*_*i *_refer to Δ_*i *_at the two levels of integration. In the case of pairwise distances between populations, *X*_*i *_and *Y*_*i *_refer to the *i*-th element of the distance matrix for each of the two levels of integration. *C *varies between -1 and +1 such that *C *= 1 for strictly positive and *C *= -1 for strictly negative covariation. It is undefined in the practically irrelevant case where a non-zero difference for one variable implies equality for the other.

### Permutation test of the significance of genetic differentiation patterns

Any increase of genetic differentiation among populations at higher levels of genetic integration is due to forces of association of genes that differ among populations. It is thus of basic interest to know whether the differentiation observed at a level of integration can be explained by random combination of genes (e.g. into diploid genotypes or haplotypes) or whether directed forces of combination must be assumed. This requires an analysis that is conditional on the gene-pool of each population, the number of populations, and the population sizes. The effects of chance can be assessed by permuting the genes within each population, such that all homologous and non-homologous combinations of genes (alleles) into (haploid, diploid or polyploid) genotypes have equal probability. For each such permutation, the values of all relevant descriptors (e.g. covariation *C *for distance matrices and differentiation snails, the mean pairwise distance Δ in the distance matrix, the symmetric population differentiation Δ_*SD*_) are determined. By performing a large number of permutations, the significance of each observed descriptor value can be measured in terms of the P-value, which is the proportion of permutations yielding descriptor values greater than or equal to the observed value. For interpretation of the results, both very small P-values (≤ 0.05) and very large P-values (≥ 0.95) are of interest.

This permutation analysis differs from common permutation analyses of differentiation among populations, in which the individuals (together with their fixed genotypes) are permuted over the populations. Such analyses aim to explain gene-pool differences among populations. In contrast, the present paper is targeted at forces of genetic differentiation that originate from the association of genes in diplo- or haplo-states and that thus go beyond those responsible for gene-pool differentiation.

## Results and discussion

### Effects of level of genetic integration on the pattern of differentiation among populations

Proceeding from lower to higher levels of integration, one expects an increase in differentiation among populations simply because of the larger varietal potential inherent in more complex structures. Since differentiation is based on distances, the distance between two populations should therefore also increase, or at least not decrease, with integration level. Consider two populations P and Q, and denote the relative frequencies of their (multilocus) genotypes at *L *(≥ 1) loci of equal degree of ploidy (≥ 1) by frequency vectors ***P ***and ***Q ***and the relative frequencies of the gene-types in their gene-pools by frequency vectors ***p ***and ***q***. Proof of the following Theorem requires the special properties of the elementary genic difference between genotypes, including the fact that it is a metric distance:

***Theorem***: *For any two populations *P*and *Q, *the distance *Δ *between the (multilocus) genetic structures ****P ****and ****Q ****at any L gene loci (L *≥ 1*) of equal degree of ploidy is not less than the mean distance between the single-locus structures ****P***^(*l*)^*and ****Q***^(*l*)^*, which in turn is not less than the distance between the corresponding gene pools ****p ****and ****q****, that is*,

$$\begin{array}{lll} \Delta (p,q) & = & \frac{1}{L}\sum_{l=1}^{L}\Delta (p^{\left(l\right)},q^{\left(l\right)})\\ & \leq & \frac{1}{L}\sum_{l=1}^{L}\Delta (P^{\left(l\right)},Q^{\left(l\right)})\\ & \leq & \Delta (P,Q) \end{array}$$

*where the difference between genetic types (haplotypes, diplotypes) is measured by the elementary genic difference d*.

*Proof*: The equality results from definition of Δ and gene-pool. The first inequality follows from Proposition 1 (see Appendix A), which states that the distance Δ between *L*-locus genotypic structures ***P ***and ***Q ***(*L *≥ 1) is never less than between the gene-pools ***p ***and ***q***. From this it follows that Δ(***p***^(*l*)^, ***q***^(*l*)^) ≤ Δ(***P***^(*l*)^, ***Q***^(*l*)^) for each locus *l*. The second inequality stems from Proposition 2 (see Appendix B), which states that the distance Δ between multilocus genotypic structures ***P ***and ***Q ***is never less than the average of the distances between the corresponding single-locus genotypic structures ***P***^(*l*) ^and ***Q***^(*l*)^.   ■

We investigated this Theorem by simulating numerous simple models. When we analyzed two populations with differing gene-pools at a locus but both showing HWP among the genotypes, we were surprised to see that the inequalities became equalities. Furthermore, the extension of HWP to inbreeding structures for the same inbreeding coefficient *F *(*i.e*., *P*_*ii *_= *p*_*i*_^2 ^+ *Fp*_*i*_(1 - *p*_*i*_) and *P*_*ij *_= 2*p*_*i*_*p*_*j*_(1 - *F*)) also yielded equality (*F *= 0 gives HWP). Equality also held when each of the genotypic structures was the product of two allelic structures (e.g., maternal and paternal), one of which was the same in both populations. When we simulated the frequencies of two-locus genotypes in two populations, both showing HWP at both loci, as the product of the single-locus genotype frequencies, equality again held. In contrast, differentiation between the gene-pool and the genotypes at a single locus did increase for inbreeding structures when the two inbreeding coefficients differed and for product structures when no two of the four allelic structures matched. No increase was obtainable between the average single-locus genotypic distance and the multilocus distance in the case of two loci, each with two alleles, not even when the selection regimes differed between the populations. It is therefore interesting that examples using real data, one of which is presented below, all showed large increases between levels, indicating that the real data does not follow simple models.

As an explanation for the examples in which the genetic distance does not increase with level of genetic integration, consider that the first inequality $\frac{1}{L}\sum_{l=1}^{L}\Delta (p^{\left(l\right)},q^{\left(l\right)})\leq \frac{1}{L}\sum_{l=1}^{L}\Delta (P^{\left(l\right)},Q^{\left(l\right)})$ becomes an equality, if Δ(***p***^(*l*)^, ***q***^(*l*)^) = Δ(***P***^(*l*)^, ***Q***^(*l*)^) holds for each single locus *l*. The calculated examples suggest that equality holds at a single locus if the genotypic structures in both populations result from the same function of their allelic structures, *i.e*., uniformity of homologous association. The second inequality $\frac{1}{L}\sum_{l=1}^{L}\Delta (P^{\left(l\right)},Q^{\left(l\right)})\leq \Delta (P,Q)$ became an equality in our calculated examples whenever multilocus genotype frequencies were the product of single-locus genotype frequencies, *i.e*., in the absence of non-homologous association.

These observations suggest that uniformity of homologous association and absence of non-homologous association result in equal distances at different integration levels. Intuitively, this coincides with the conception that absence or uniformity of association do not really introduce any new structure to the higher levels of integration. Since this phenomenon only shows up when the difference between genotypes is measured by the elementary genic distance, this measure is closely tied to the concept that the absence of association does not lead to higher differentiation at higher levels of genetic integration.

Nevertheless, absence of non-homologous association may not be a necessary condition for equality, since $\frac{1}{L}\sum_{l=1}^{L}\Delta (P^{\left(l\right)},Q^{\left(l\right)})=\Delta (P,Q)$ also occurred in some examples where association between loci was present. This means that the basic prerequisite for validity of Δ(***p***, ***q***) = Δ(***P***, ***Q***) (stated at the end of Appendix A), namely that every gene-type that is not of equal frequency in the two populations be either a source gene or a sink gene, may be fulfilled even in the presence of non-homologous association.

Carrying these results for Δ over to the differentiation measures Δ_*j *_and Δ_*SD*_, the differentiation among populations for multilocus genetic types (haplotypes, genotypes) equals the gene-pool differentiation if all populations show uniformity of homologous gene association (e.g. HWP, inbreeding for the same inbreeding coefficient) and absence of non-homologous association. Otherwise, differentiation may increase with level of integration, as expected.

All of these results are based on the special measure of elementary genic difference between genotypes (for any degree of ploidy). Thus any other measure is likely to yield different results, the interpretation of which would of course depend on a clear conceptual understanding of the difference measure. In particular, this concerns genetic associations that are not specifically genic. A discussion of these measures (see [21] for an overview of measures) would, however, be clearly beyond the scope of this paper.

### Application of the approach to an assemblage of oak stands

The effects of the level of genetic integration on patterns of differentiation will be illustrated with the help of an example based on published data [20,22]. The reason for not applying it to particular models here is to show how insights can be gained directly from observations, without model constraints. In this data, the multilocus genotypes at the same six nuclear microsatellite loci were scored in all adult trees of four stands of pedunculate oak (*Quercus robur*) located in north-central Germany. Of the 159 trees in the stand near Rantzau, 154 trees could be scored at all six loci, yielding 153 different multilocus genotypes (abbreviated 159/154/153). The other three stands are near Behlendorf (228/178/177), Steinhorst (85/74/74), and Escherode (210/200/200). The number of alleles per locus lies between 15 and 35 with a mean of 23.7, of which an average of five occur in only one stand. Each multilocus genotype appeared in only one stand, yielding a total of 604 different genotypes among the 606 trees scored at all loci. Failure to score the complete multilocus genotypes of the other 76 trees in the stands is assumed to be independent of their genotypes.

Table 1 lists the distance matrix of pairwise distances Δ between stands and their mean as well as the symmetric population differentiation Δ_*SD *_and its components Δ_*j*_, both based on the elementary genic difference between genetic types, for each of three levels of integration: the gene-pool distance is the average of the six single-locus allelic distances; the single-locus diplophase distance is also the average over the loci; the multilocus diplophase distance. It is seen that for each pair of stands, all pairwise distances Δ increase considerably with the level of integration. This indicates that neither the gene association within single loci (homologous association) nor the gene association among loci (non-homologous association) is of the same form in any two stands, and in particular that association is present. Both the distances and the snail components show a much larger increase between the single-locus diplophase and the multilocus diplophase than between the gene-pool and the single-locus diplophase. Hence the non-homologous gene associations make a distinctly greater contribution to the differentiation than the homologous gene associations. It is interesting to consider the large increase between the single-locus and the multilocus level in the light of our failure to produce any increase at all when simulating simple selection models, as mentioned above. This indication that the data is not explainable by simple models requires further investigation.

**Table 1:** Genetic differentiation among four oak stands at three levels of genetic integration.

**Genetic differentiation among stands for three levels of integration**						
Level of integration	Genetic distance between stands				Components of the differentiation snail	
Gene-pool (GP)	Δ	R	B	S	Δ_R_	0.137
	B	0.158			Δ_B_	0.129
	S	0.182	0.163		Δ_S_	0.148
	E	0.157	0.172	0.171	Δ_E_	0.143
	
Mean	0.167				Δ_SD_	0.139

Single-locus diplophase (SLD)	Δ	R	B	S	Δ_R_	0.208
	B	0.226			Δ_B_	0.185
	S	0.261	0.234		Δ_S_	0.224
	E	0.217	0.222	0.234	Δ_E_	0.186
	
Mean	0.232 [0.214, 0.235] 0.004 ↑ **				Δ_SD_	0.200 [0.184, 0.203] 0.002 ↑ **

Multilocus diplophase (MLD)	Δ	R	B	S	Δ_R_	0.501
	B	0.510			Δ_B_	0.495
	S	0.540	0.531		Δ_S_	0.523
	E	0.502	0.503	0.522	Δ_E_	0.487
	
Mean	0.518 [0.507, 0.521] 0.005 ↑ **				Δ_SD_	0.502 [0.489, 0.505] 0.006 ↑ **

**Covariation of genetic differentiation between integration levels**						
Comparison	Genetic distances				Differentiation snail	

GP vs. SLD	0.893 [0.421, 0.988] 0.270 n.s.				0.809 [0.545, 1.000] 0.912 n.s.	
SLD vs. MLD	1.000 [0.742, 1.000] 0.084 n.s.				0.995 [0.868, 1.000] 0.532 n.s.	
GP vs. MLD	0.720 [0.395, 0.954] 0.799 n.s.				0.657 [0.376, 1.000] 0.965 ↓ *	

For four stands (abbr. R, B, S, and E) of pedunculate oak in north-central Germany scored at six nuclear microsatellite loci, genetic differentiation based on the elementary genic difference between genetic types was calculated at three levels of genetic integration: gene-pool, single-locus diplophase, and multilocus diplophase. The observed genetic distance Δ between each pair of stands and the observed distance Δ_*j *_(component of differentiation snail) of each stand *j *from its complement are listed together with their respective means. To assess the effect of integration level on patterns of differentiation, the lower part of the table shows the covariation between integration levels of the pairwise genetic distances Δ and of the snail components Δ_*j*_. To compare the observed distances with those obtainable if the genes were randomly arranged, 10 000 data sets were generated by random permutation of the genes at each locus within (not among) all stands. Square brackets enclose the ranges [min, max] of 10 000 distances by permutation, followed by the P-values (*i.e*., proportion of permutations yielding distances equal to or greater than the observed distance). Symbols ↑ ** and ↓ * indicate that fewer than 1% and more than 95%, respectively, of the permutations yielded distances equal to or greater than the observed distance

In order to be sure that this apparent discrepancy between stands in the form of association is not simply due to the small number of multilocus genotypes in the stands compared to the number that could be formed from the genes present in the stands, a permutation analysis was performed as described above. Ten thousand new data sets were generated by random permutation of the genes at each locus within each stand to form new single-locus genotypes, randomly combined to multilocus genotypes. Each observed distance was then compared to the 10 000 distances from permutation. Surprisingly, for both the single-locus diplophase and the multilocus diplophase, the observed mean pairwise distance and the symmetric population differentiation Δ_*SD *_were significantly high (*i.e*., higher than for 99% of all permutations). This indicates that both homologous and non-homologous association of genes follow very different rules among the stands.

The significant size of the mean of the pairwise distances for the single-locus diplophase and the multilocus diplophase may seem counterintuitive to the striking similarity of these distances within each of the three levels of integration. The same holds for the snail components. To explain this similarity, note that the range of values that appeared in the permutations is also quite narrow. Thus the collections of genes in the stands must place tight limits on the achievable distances and snail components.

Not only the sizes but also the covariation *C *of the pairwise distances Δ and the snail components Δ_*j *_at the different integration levels depend on the differences in gene association between levels. The positive covariation of distance matrices and of snail components for all pairs of integration levels shows that no form of association completely overturns the ranking prescribed by the gene-pool. Whereas the gene arrangements that distinguish the single-locus diplophase from the gene-pool do produce rank changes among the stands (*C *= 0.893 for the distance matrix and *C *= 0.809 for the snail components), the gene arrangements that distinguish the single-locus diplophase from the multilocus diplophase have little effect on ranking (*C *= 1 for the distance matrix and *C *= 0.995 for the snail components). Not surprisingly, the gene arrangements that distinguish the gene-pool from the multilocus diplophase yield the weakest covariation (*C *= 0.720 for the distance matrix and *C *= 0.657 for the snail components).

**Figure 1:**
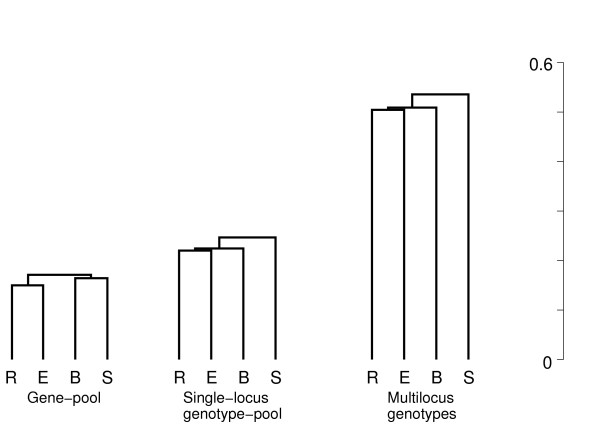
**UPGMA dendrograms at three levels of genetic integration in four oak stands**. For six microsatellite loci scored in four stands of oak (R, B, S, E), UPGMA dendrograms were constructed from the matrices of genetic distances Δ between stands in Tab. 1. Within each dendrogram, the quantitative differences between clusters are weak. The gene-pool dendrogram differs qualitatively, *i.e*., topologically, from the topologically identical dendrograms of the higher levels. The significantly large increase in the mean pairwise distance, and thus in the length of the dendrograms, with level of integration implies that the stands show differentiation for their forms of homologous gene association and, even more so, non-homologous association.

**Figure 2:**
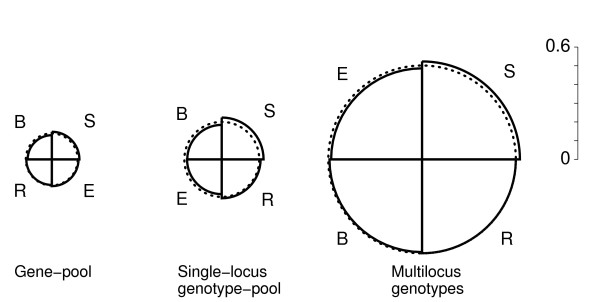
**Differentiation snails at three levels of genetic integration in four oak stands**. For six microsatellite loci scored in four stands of oak (R, B, S, E) the differentiation snails were constructed from the snail components Δ_*j *_in Tab. 1. Dotted circles mark the symmetric population differentiation Δ_*SD*_. Within each snail, the quantitative differences among the components are slight. Each snail differs qualitatively in the ranking of the stands from the other two (*i.e*., covariation *C *< 1 for each comparison). The significantly large increase in the radius Δ_*SD *_of the snails with each higher integration level confirms the differentiation among the stands for form of gene association.

This pattern of strong covariation is evident in the UPGMA dendrograms (Fig. 1) based on the three distance matrices, which are easier to visualize than the distance matrices themselves, and the differentiation snails (Fig. 2) constructed from the three sets of snail components. The dendrograms show weakly defined clusters that vary in topology between the gene-pool and the topologically identical clusters of the single-locus diplophase and the multilocus diplophase. The snails show rank changes that are based on only slight differences between the snail components.

It is interesting to compare the observed covariations with the ranges of covariation that occurred for the gene arrangements generated by the 10 000 random permutations. The distance matrices show weaker covariation between the single-locus diplophase and the multilocus diplophase in almost 92% of the permutations (P-value 0.084 for *C *= 1) but between the gene-pool and the single-locus diplophase for only 73% (P-value 0.270 for *C *= 0.893). From the high improbability of the observed perfect covariation (*C *= 1) between the single-locus diplophase and the multilocus diplophase, it can be inferred that the non-homologous association has a special relationship to the homologous association in the single-locus diplophase. In contrast, the intermediate P-value for the covariation between the gene-pool and the single-locus diplophase implies that the homologous association is not predetermined by the collection of genes.

The snail components showed a weaker covariation between the single-locus diplophase and the multilocus diplophase for ca. 47% of the permutations (P-value 0.532 for *C *= 0.995) but between the gene-pool and the single-locus diplophase only for ca. 9% (P-value 0.912 for *C *= 0.809). This confirms the stronger effect of homologous association than non-homologous association on the ranking within the distance matrices. Compared to these, however, the snail components show stronger covariation than observed for a much higher proportion of the permutations, both for homologous and non-homologous association. Hence, the covariation of the snail components seems to be less sensitive to the effects of gene association than is the covariation of the pairwise distances. This must be due to the equalizing influence of combining three stands for comparison to the fourth that is the basis of the snail components.

### Discussion of the application to the oak stands

The differentiation observed among the oak stands increases distinctly from the gene-pool level to the single-locus diplophase. An even larger jump in differentiation occurs when the non-homologous association for the multiple loci is included. These are clear indications that all (except for perhaps one) of the stands show deviation from both HWP and gametic equilibrium, and that the degrees of deviation vary considerably among the stands. Such indications could not be confirmed by conventional statistical testing due to the large numbers of degrees of freedom and the implied weakness of the respective test statistics. It might come as a surprise that the application of the special permutation analysis presented above to genetic differences *between *populations detects association characteristics *within *populations. Confirmation and exploitation of this statistical potential deserves further investigation.

Consequently, if the four oak stands had been less clearly separated spatially, and if we had wanted to assign the trees to their proper subpopulations, we would have run into problems when making use of methods based on the absence of gene associations within populations. Methods for finding subdivisions of populations that are based on Hardy-Weinberg proportions and gametic equilibrium within populations (e.g. [23-27]) may therefore not have assigned the individuals to their original stands.

When comparing the observed differentiation to that producible by gene association in the stands, all 10 000 permutations agreed with the observation by showing much higher differentiation among the single-locus diplophases than among the gene-pools, both for the mean pairwise genetic distance and the symmetric population differentiation Δ_*SD*_. This tells us not only that the random generation of gene association never yielded Hardy-Weinberg structures for all loci in all four stands simultaneously. Neither was any other form of homologous association realized simultaneously that leaves differentiation unchanged (e.g. inbreeding with equal coefficients). Furthermore, all non-homologous associations showed a considerable additional increase in differentiation over the homologous associations, as is seen in the wide separation of the range of differentiation for the single-locus diplophase from the range for the multilocus diplophase. Remarkably, both ranges of differentiation are quite narrow. These results indicate that the increases in differentiation that are realizable by homologous and non-homologous gene association can be tightly restricted by the genic composition of the populations. In such cases, equal differentiation at consecutive integration levels may not be achievable. Thus it appears that differentiation among populations with respect to their forms of gene association may be a normal occurrence. This insight questions the common practice of restricting the measurement of population differentiation to the allelic level (e.g. *F*_*ST*_), thereby ignoring the considerable effects of gene association on population differentiation. This analysis is the first of its kind. Therefore, we cannot venture a prediction about whether the above findings on covariation between levels of integration constitute a general trend. It is conceivable, for example, that these findings are mainly determined by the conspicuously large polymorphism characteristic of the microsatellite markers used in this study. Other genetic markers may tell different stories.

## Conclusion

This new approach to the analysis of genetic differentiation among populations demonstrates that the consideration of gene associations within populations adds a new quality to studies on population differentiation that is overlooked when viewing only gene-pools.

## Appendix A

***Proposition 1***: *For any two populations P and Q, the distance between the (multilocus) genetic structures **P **and **Q **at any L gene loci (L *≥ 1*) of equal degree of ploidy is not less than the distance between the corresponding gene pools ****p ****and ****q****, respectively, that is,*

$$\Delta (p,q)=\frac{1}{L}\sum_{l=1}^{L}d_{0}(p^{\left(l\right)},q^{\left(l\right)})\leq \Delta (P,Q)$$

*where the difference between genetic types (haplotypes, diplotypes) is measured by the elementary genic difference d*.

Whereas the equality in Proposition 1 follows from the text, proof of the inequality in Proposition 1 depends on a lemma that applies the following notation: For two populations P and Q, let *G*_*x *_or *G*_*y *_denote the genetic types of the individuals at *L *gene loci of degree of ploidy *N = *1, yielding *K *= *LN *genes per individual. For the relative frequencies *P*(*G*_*x*_) and *Q*(*G*_*x*_) of type *G*_*x *_in the two populations (by some ordering), denote the frequency structure of the *L*-locus types as ***P ***and ***Q***. Call the *i*th allele at the *l*-th locus *A*_*i*;*l*_. Term the frequency structure of the gene-types *A*_*i*;*l *_in the *L*-locus gene-pool as ***p ***and ***q***. A shift transformation *s*(***P***, ***Q***) decomposes the set of all genetic types on the basis of their relative frequencies into three sets: The *source types G*_*x *_for which *P *(*G*_*x*_) > *Q*(*G*_*x*_) holds, *i.e*., that show an excess in the first population with respect to the second; the *sink types G*_*x *_for which *P *(*G*_*x*_) <*Q*(*G*_*x*_) holds, *i.e*., that show a deficit in the first population; and those for which *P*(*G*_*x*_) = *Q*(*G*_*x*_) holds. In general terms, the excess of type *G*_*x *_is quantifiable as *P *(*G*_*x*_) - min{*P *(*G*_*x*_), *Q*(*G*_*x*_)} ≥ 0, with equality to 0 if *P*(*G*_*x*_) ≤ *Q*(*G*_*x*_). Likewise, the deficit of type *G*_*x *_is quantifiable as *Q*(*G*_*x*_) - min{*P *(*G*_*x*_), *Q*(*Gx*)} ≥ 0, with equality to 0 if *P*(*G*_*x*_) ≥ *Q*(*G*_*x*_). For all types *G*_*x*_, *s*(***P***, ***Q***) fulfills:

$$\begin{array}{lll} s(G_{x},G_{x}) & = & 0\\ s(G_{x},\cdot ) & := & \sum_{y}s(G_{x},G_{y})\\ & = & P(G_{x})-\min\{P(G_{x}),Q(G_{x})\}\\ s(\cdot ,G_{x}) & := & \sum_{y}s(G_{y},G_{x})\\ & = & Q(G_{x})-\min\{P(G_{x}),Q(G_{x})\} \end{array}$$

where: *s*(*G*_*x*_, *G*_*y*_) is the relative frequency among all individuals in population P of individuals that are shifted from type *G*_*x *_to type *G*_*y*_.

***Lemma 1***: *Consider any shift transformation s*(***P***, ***Q***) *between the L-locus genetic structures. The genetic distance *$d_{0}(p^{\left(l\right)},q^{\left(l\right)}):=\frac{1}{2}\sum_{i}\left|p_{i}^{\left(l\right)}-q_{i}^{\left(l\right)}\right|$*between the corresponding allelic structures ****p***^(*l*) ^*and ****q***^(*l*)^* at locus l is expressible as:*

$$d_{0}(p^{\left(l\right)},q^{\left(l\right)})=\frac{1}{2}\sum_{i}|\;\alpha (A_{i;l,}\cdot )-\alpha (\cdot ,A_{i;l})|$$

*where: *$p_{i}^{\left(l\right)}$*and *$q_{i}^{\left(l\right)}$*is the relative frequency of allele A*_*i*;*l *_*at locus l in population *P*and *Q*, respectively, where the α are defined as:*

$$\begin{array}{lllll} \alpha (A_{i;l,}\cdot ) & := & \frac{1}{N}\sum_{x}n_{i;l}(G_{x})\cdot s(G_{x},\cdot ) & = & \frac{1}{N}\sum_{x,y}n_{i;l}(G_{x})\cdot s(G_{x},G_{y})\\ \alpha (\cdot ,A_{i;l}) & := & \frac{1}{N}\sum_{x}n_{i;l}(G_{x})\cdot s(\cdot ,G_{x}) & = & \frac{1}{N}\sum_{x,y}n_{i;l}(G_{y})\cdot s(G_{x},G_{y}) \end{array}$$

*and where n*_*i*;*l*_(*G*_*x*_) *is the number of genes of allelic type A*_*i*;*l *_*in type G*_*x*_.

*Proof*: Note that since an allele *A*_*i*;*l *_can be present in both source and sink types, *α*(*A*_*i*;*l*_, •) > 0 and *α*(•, *A*_*i*;*l*_) > 0 can hold simultaneously. It follows that

$$\begin{array}{l} \frac{1}{2}\sum_{i}\left|\alpha (A_{i;l,}\cdot )-\alpha (\cdot ,A_{i;l})\right|\\ =\frac{1}{2}\sum_{i}\left|\frac{1}{N}\sum_{x}n_{i;l}(G_{x})\cdot s(G_{x},\cdot )-\frac{1}{N}\sum_{x}n_{i;l}(G_{x})\cdot s(\cdot ,G_{x})\right|\\ =\frac{1}{2}\sum_{i}|\left(\frac{1}{N}\sum_{x}n_{i;l}(G_{x})\cdot \left[P(G_{x})-\min\{P(G_{x}),Q(G_{x})\}\right]\right)\\ -\left(\frac{1}{N}\sum_{x}n_{i;l}(G_{x})\cdot \left[Q(G_{x})-\min\{P(G_{x}),Q(G_{x})\}\right]\right)|\\ =\frac{1}{2}\sum_{i}\left|\frac{1}{N}\sum_{x}n_{i;l}(G_{x})\cdot \left[P(G_{x})-Q(G_{x})\right]\right|\\ =\frac{1}{2}\sum_{i}\left|p_{i}^{\left(l\right)}-q_{i}^{\left(l\right)}\right|\\ =d_{0}(p^{\left(l\right)},q^{\left(l\right)}) \end{array}$$

■

Note that *s*(*G*_*x*_, *G*_*y*_) > 0 is true only if *G*_*x *_is a source type and *G*_*y *_a sink type. Thus *α*(*A*_*i*;*l*_, •) quantifies the total number of *A*_*i*;*l*_-genes in the original (source) types of all shifted individuals, divided by the total number of genes at locus *l *in Population P(= *N· *population size). Analogously, *α*(•, *A*_*i*;*l*_) quantifies the number of *A*_*i*;*l*_-genes in the new (sink) types of all shifted individuals, divided by the same total number of genes. Their difference is the net frequency with which this allele was shifted.

***Proof of Proposition 1***: For any shift transformation *s*(***P***, ***Q***), it follows from Lemma 1 and the definition of the *α *that:

$$\begin{array}{ll} & \frac{1}{L}\sum_{l=1}^{L}d_{0}(p^{\left(l\right)},q^{\left(l\right)})\\ = & \frac{1}{L}\sum_{l=1}^{L}\frac{1}{2}\sum_{i}\left|\alpha (A_{i;l},\cdot )-\alpha (\cdot ,A_{i;l})\right|\\ = & \frac{1}{L}\sum_{l=1}^{L}\frac{1}{2}\sum_{i}\left|\frac{1}{N}\sum_{x,y}\left(n_{i;l}(G_{x})-n_{i;l}(G_{y})\right)\cdot s(G_{x},G_{y})\right|\\ \leq & \frac{1}{L}\sum_{l=1}^{L}\frac{1}{2}\sum_{i}\frac{1}{N}\sum_{x,y}\left|n_{i;l}(G_{x})-n_{i;l}(G_{y})\right|\cdot s(G_{x},G_{y})\\ = & \sum_{x,y}\left(\frac{1}{2LN}\sum_{l=1}^{L}\sum_{i}\left|n_{i;l}(G_{x})-n_{i;l}(G_{y})\right|\right)\cdot s(G_{x},G_{y})\\ = & \sum_{x,y}d(G_{x},G_{y})\cdot s(G_{x},G_{y}) \end{array}$$

The final equality follows from the definition of *d*(*G*_*x*_, *G*_*y*_) in the text. Since this holds for any shift transformation, it also holds if *s*(***P***, ***Q***) is a minimum shift transformation, in which case ∑_*x*,*y*_*d*(*G*_*x*_, *G*_*y*_)·*s*(*G*_*x*_, *G*_*y*_) = Δ(***P***, ***Q***). Therefore, it follows that: $\Delta (p,q)=\frac{1}{L}\sum_{l=1}^{L}d_{0}(p^{\left(l\right)},q^{\left(l\right)})\leq \Delta (P,Q)$, as claimed.   ■

In Proposition 1, equality holds if and only if for each gene-type *A*_*i*;*l*_, the expression

(*n*_*i*;*l*_(*G*_*x*_) - *n*_*i*;*l*_(*G*_*y*_))·*s*(*G*_*x*_, *G*_*y*_)

has the same sign for all pairs of types *G*_*x*_, *G*_*y*_. This distinguishes three special groups of genes: Genes *A*_*i*;*l *_for which the expression equals zero for all pairs of types *G*_*x*_, *G*_*y*_, implying that *A*_*i*;*l *_is equally frequent in the two populations and therefore shows no net shift; genes *A*_*i*;*l *_for which the expression is ≥ 0 but not ≡ 0 for all *x*, *y*, that is, that are never less frequent in source types *G*_*x *_than in the corresponding sink types *G*_*y*_, making them *source genes*; genes *A*_*i*;*l *_for which the expression is ≤ 0 but not ≡ 0 for all *x*, *y*, making them *sink genes*. (Note that a gene need not belong to any of the three groups, as is demonstrated by *s*(*A*_*i*;*l*_*A*_*j*;*l*_, *A*_*j*;*l*_*A*_*j*;*l*_) > 0 and *s*(*A*_*i*;*l*_*A*_*j*;*l*_, *A*_*i*;*l*_*A*_*i*;*l*_) > 0.)

## Appendix B

***Proposition 2***: *For any two populations *P*and *Q*, the distance between the (multilocus) genetic structures ****P ****and ****Q ****at any L gene loci (L ≥ *1*) of equal degree of ploidy N ≥ *1 *is not less than the mean distance between the corresponding single-locus structures ****P***^(*l*) ^*and ****Q***^(*l*)^, *respectively, that is*,

$$\frac{1}{L}\sum_{l=1}^{L}\Delta (P^{\left(l\right)},Q^{\left(l\right)})\leq \Delta (P,Q)$$

*where the difference between genetic types is measured by the elementary genic difference d*.

The validity of Proposition 2 for *L *= 1 is obvious. For *L *≥ 2, proof depends on four lemmata that apply the following notation: Let *s*(***P***, ***Q***) be a shift transformation between the *L*-locus genotypic structures. Denote the various *L*-locus types as *G*_*x *_or *G*_*y*_, and write each type *G*_*x *_as the "product" $G_{x}^{\left(l\right)}G_{x}^{\left(\{1,...,L\}\backslash l\right)}$ of its projection $G_{x}^{\left(l\right)}$ to the single-locus type at loci *l *= 1 and its projection $G_{x}^{C(l)}$ to the complementary (*L *- 1)-locus type. Denote the single-locus types at locus *l *as $g_{u}^{\left(l\right)}$ or $g_{u}^{\left(l\right)}$ and the complementary types as $g_{u}^{C(l)}$ or $g_{v}^{C(l)}$. Define

$$\mu_{l}(g_{u}^{\left(l\right)},g_{v}^{\left(l\right)}):=\sum_{\left\{x|G_{x}^{\left(l\right)}=g_{u}^{\left(l\right)}\right\}}\sum_{\left\{y|G_{y}^{\left(l\right)}=g_{v}^{\left(l\right)}\right\}}s(G_{x},G_{y})$$

as the marginal sum of all shifts that involve the type $g_{u}^{\left(l\right)}$ at locus *l *in the source type *G*_*x *_and $g_{u}^{\left(l\right)}$ in the sink type *G*_*y*_.

***Lemma 2 ****The difference *$\sum_{v}\mu_{l}(g_{u}^{\left(l\right)},g_{v}^{\left(l\right)})-\sum_{v}\mu_{l}(g_{v}^{\left(l\right)},g_{u}^{\left(l\right)})$*between the marginal sums for any u equals the net shift *$\sum_{v}s_{l}(g_{u}^{\left(l\right)},g_{v}^{\left(l\right)})-\sum_{v}s_{l}(g_{v}^{\left(l\right)},g_{u}^{\left(l\right)})$* for any shift transformation s*_*l *_*at the locus*.

*Proof*: For the *l*-th locus it holds that:

$$\begin{array}{ll} & \sum_{v}\mu_{l}(g_{u}^{\left(l\right)},g_{v}^{\left(l\right)})\\ = & \sum_{u,t,w}s(g_{u}^{\left(l\right)}g_{t}^{C(l)},g_{v}^{\left(l\right)}g_{w}^{C(l)})\\ = & \sum_{t}\left[P(g_{u}^{\left(l\right)}g_{t}^{C(l)})-\min\left\{P(g_{u}^{\left(l\right)}g_{t}^{C(l)}),Q(g_{u}^{\left(l\right)}g_{t}^{C(l)})\right\}\right]\\ & \sum_{v}\mu_{l}(g_{v}^{\left(l\right)},g_{u}^{\left(l\right)})\\ = & \sum_{v,t,w}s(g_{v}^{\left(l\right)}g_{w}^{C(l)},g_{u}^{\left(l\right)}g_{t}^{C(l)})\\ = & \sum_{t}\left[Q(g_{u}^{\left(l\right)}g_{t}^{C(l)})-\min\left\{P(g_{u}^{\left(l\right)}g_{t}^{C(l)}),Q(g_{u}^{\left(l\right)}g_{t}^{C(l)})\right\}\right] \end{array}$$

Their difference equals:

$$\sum_{t}P(g_{u}^{\left(l\right)}g_{t}^{C(l)})-\sum_{t}Q(g_{u}^{\left(l\right)}g_{t}^{C(l)})=P(g_{u}^{\left(l\right)})-Q(g_{u}^{\left(l\right)})$$

The same difference results for any shift transformation *s*_*l *_at a locus *l*, since:

$$\begin{array}{l} \sum_{v}s_{l}(g_{u}^{\left(l\right)},g_{v}^{\left(l\right)})=P^{\left(l\right)}(g_{u}^{\left(l\right)})-\min\{P^{\left(l\right)}(g_{u}^{\left(l\right)}),Q^{\left(l\right)}(g_{u}^{\left(l\right)})\}\\ \sum_{v}s_{l}(g_{v}^{\left(l\right)},g_{u}^{\left(l\right)})=Q^{\left(l\right)}(g_{u}^{\left(l\right)})-\min\{P^{\left(l\right)}(g_{u}^{\left(l\right)}),Q^{\left(l\right)}(g_{u}^{\left(l\right)})\} \end{array}$$

■

Even though marginal sums share this property with any shift transformation at the locus, the following lemma shows that marginal sums may not specify a shift transformation.

***Lemma 3***: *The marginal sums *$\mu_{l}(g_{u}^{\left(l\right)},g_{v}^{\left(l\right)})$* of all types *$g_{u}^{\left(l\right)}$, $g_{u}^{\left(l\right)}$* at locus l may shift an amount that is in excess of the amount required of any shift transformation at the locus*.

*Proof*: The total amount shifted away from any type $g_{u}^{\left(l\right)}$ at locus *l *equals

$$\begin{array}{ll} & \sum_{v}\mu_{l}(g_{u}^{\left(l\right)},g_{v}^{\left(l\right)})\\ = & P^{\left(l\right)}(g_{u}^{\left(l\right)})-\sum_{t}\min\left\{P(g_{u}^{\left(l\right)}g_{t}^{C(l)}),Q(g_{u}^{\left(l\right)}g_{t}^{C(l)})\right\}\\ \geq & P^{\left(l\right)}(g_{u}^{\left(l\right)})-\min\left\{\sum_{t}P(g_{u}^{\left(l\right)}g_{t}^{C(l)}),\sum_{t}Q(g_{u}^{\left(l\right)}g_{t}^{C(l)})\right\}\\ = & P^{\left(l\right)}(g_{u}^{\left(l\right)})-\min\left\{P^{\left(l\right)}(g_{u}^{\left(l\right)}),Q^{\left(l\right)}(g_{u}^{\left(l\right)})\right\} \end{array}$$

By the same reasoning, the amount received by $g_{u}^{\left(l\right)}$ equals

$$\sum_{v}\mu_{l}(g_{v}^{\left(l\right)},g_{u}^{\left(l\right)})\geq Q^{\left(l\right)}(g_{u}^{\left(l\right)})-\min\left\{P^{\left(l\right)}(g_{u}^{\left(l\right)}),Q^{\left(l\right)}(g_{u}^{\left(l\right)})\right\}$$

These inequalities contradict the equality required of a shift transformation.   ■

Lemma 3 shows that the marginal sums may shift too much, and it is easy to construct examples for which this is the case. Excess amounts must be due to the appearance of one or more single-locus types both in two-locus source types and in two-locus sink types. This makes them both sources and sinks in the marginal sums, in violation of the properties of a shift transformation. The three ways in which a type $g_{a}^{\left(l\right)}$ can act as both a source and a sink are:

$$\begin{array}{l} \begin{array}{cc} Case\;1: & \mu_{l}(g_{a}^{\left(l\right)},g_{a}^{\left(l\right)})>0 \end{array}\\ \begin{array}{ccccc} Case\;2: & \mu_{l}(g_{a}^{\left(l\right)},g_{b}^{\left(l\right)})>0 & \text{and} & \mu_{l}(g_{b}^{\left(l\right)},g_{a}^{\left(l\right)})>0 & \left(a\neq b\right) \end{array}\\ \begin{array}{ccccccccc} Case\;3: & \mu_{l}(g_{a}^{\left(l\right)},g_{b}^{\left(l\right)})>0 & \text{and} & \mu_{l}(g_{b}^{\left(l\right)},g_{c}^{\left(l\right)})>0 & (a\neq b & \text{and} & b\neq c & \text{and} & a\neq c) \end{array} \end{array}$$

The following lemma shows how to eliminate all ambivalent source/sink relationships from the marginal sums without changing the net amount shifted, *i.e*., amount sent away as a source minus the amount received as a sink.

***Lemma 4***: *The marginal sums *$\mu_{l}(g_{u}^{\left(l\right)},g_{v}^{\left(l\right)})$* of all types *$g_{u}^{\left(l\right)}$, $g_{u}^{\left(l\right)}$* at locus l can be used to construct a quasi-shift κ*_*l*_(***P***^(*l*)^, ***Q***^(*l*)^) *with the following three properties:*

$$\begin{array}{c} \sum_{u,v}d(g_{u}^{\left(l\right)},g_{v}^{\left(l\right)})\cdot \mu_{l}(g_{u}^{\left(l\right)},g_{v}^{\left(l\right)})\geq \sum_{u,v}d(g_{u}^{\left(l\right)},g_{v}^{\left(l\right)})\cdot \kappa_{l}(g_{u}^{\left(l\right)},g_{v}^{\left(l\right)})\\ \sum_{v}\kappa_{l}(g_{u}^{\left(l\right)},g_{v}^{\left(l\right)})\cdot \sum_{v}\kappa_{l}(g_{v}^{\left(l\right)},g_{u}^{\left(l\right)})=0\\ \sum_{v}\mu_{l}(g_{u}^{\left(l\right)},g_{v}^{\left(l\right)})-\sum_{v}\mu_{l}(g_{v}^{\left(l\right)},g_{u}^{\left(l\right)})=\sum_{v}\kappa_{l}(g_{u}^{\left(l\right)},g_{v}^{\left(l\right)})-\sum_{v}\kappa_{l}(g_{v}^{\left(l\right)},g_{u}^{\left(l\right)})=P(g_{u}^{\left(l\right)})-Q(g_{u}^{\left(l\right)}) \end{array}$$

*Proof by construction*: Consider the following algorithm:

*START: *Set $\kappa_{l}(g_{u}^{\left(l\right)},g_{v}^{\left(l\right)})\leftarrow \mu_{l}(g_{u}^{\left(l\right)},g_{v}^{\left(l\right)})$ for all *u*, *v*.

*Step 1: *If $\kappa_{l}(g_{a}^{\left(l\right)},g_{a}^{\left(l\right)})>0$ holds for a type $g_{a}^{\left(l\right)}$, set $\kappa_{l}(g_{a}^{\left(l\right)},g_{a}^{\left(l\right)})\leftarrow 0$. Since $d(g_{a}^{\left(l\right)},g_{a}^{\left(l\right)})=0$, this has no effect on the sum $\sum_{u,v}d(g_{u}^{\left(l\right)},g_{v}^{\left(l\right)})\cdot \mu_{l}(g_{u}^{\left(l\right)},g_{v}^{\left(l\right)})$. Repeat for an additional type fulfilling the condition. If none exist, go to Step 2.

*Step 2: *If $\kappa_{l}(g_{a}^{\left(l\right)},g_{b}^{\left(l\right)})>0$ and $\kappa_{l}(g_{b}^{\left(l\right)},g_{a}^{\left(l\right)})>0$ hold for *a *≠*b*, set

$$\begin{array}{lll} {\kappa^{\prime }}_{l}(g_{a}^{\left(l\right)},g_{b}^{\left(l\right)}) & \leftarrow & \kappa_{l}(g_{a}^{\left(l\right)},g_{b}^{\left(l\right)})-M\\ {\kappa^{\prime }}_{l}(g_{b}^{\left(l\right)},g_{a}^{\left(l\right)}) & \leftarrow & \kappa_{l}(g_{b}^{\left(l\right)},g_{a}^{\left(l\right)})-M \end{array}$$

where $M:=\min\left\{\kappa_{l}(g_{a}^{\left(l\right)},g_{b}^{\left(l\right)}),\kappa_{l}(g_{b}^{\left(l\right)},g_{a}^{\left(l\right)})\right\}$. Because

$$\begin{array}{ll} & d(g_{a}^{\left(l\right)},g_{b}^{\left(l\right)})\cdot {\kappa^{\prime }}_{l}(g_{a}^{\left(l\right)},g_{b}^{\left(l\right)})+d(g_{b}^{\left(l\right)},g_{a}^{\left(l\right)})\cdot {\kappa^{\prime }}_{l}(g_{b}^{\left(l\right)},g_{a}^{\left(l\right)})\\ = & d(g_{a}^{\left(l\right)},g_{b}^{\left(l\right)})\cdot \left[\kappa_{l}(g_{a}^{\left(l\right)},g_{b}^{\left(l\right)})+\kappa_{l}(g_{b}^{\left(l\right)},g_{a}^{\left(l\right)})-2\cdot M\right]\\ \leq & d(g_{a}^{\left(l\right)},g_{b}^{\left(l\right)})\cdot \kappa_{l}(g_{a}^{\left(l\right)},g_{b}^{\left(l\right)})+d(g_{b}^{\left(l\right)},g_{a}^{\left(l\right)})\cdot \kappa_{l}(g_{b}^{\left(l\right)},g_{a}^{\left(l\right)}) \end{array}$$

it follows that

$$\sum_{u,v}d(g_{u}^{\left(l\right)},g_{v}^{\left(l\right)})\cdot \kappa_{l}(g_{u}^{\left(l\right)},g_{v}^{\left(l\right)})\geq \sum_{u,v}d(g_{u}^{\left(l\right)},g_{v}^{\left(l\right)})\cdot {\kappa^{\prime }}_{l}(g_{u}^{\left(l\right)},g_{v}^{\left(l\right)})$$

Set

$$\begin{array}{lll} \kappa_{l}(g_{a}^{\left(l\right)},g_{b}^{\left(l\right)}) & \leftarrow & {\kappa^{\prime }}_{l}(g_{a}^{\left(l\right)},g_{b}^{\left(l\right)})\\ \kappa_{l}(g_{b}^{\left(l\right)},g_{a}^{\left(l\right)}) & \leftarrow & {\kappa^{\prime }}_{l}(g_{b}^{\left(l\right)},g_{a}^{\left(l\right)}) \end{array}$$

Repeat for an additional pair of types that fulfill the condition. If none exist, go to Step 3.

*Step 3: *If $\kappa_{l}(g_{a}^{\left(l\right)},g_{b}^{\left(l\right)})>0$ and $\kappa_{l}(g_{b}^{\left(l\right)},g_{c}^{\left(l\right)})>0$ hold for three different indices *a*, *b*, *c, *subtract $M:=\min\left\{\kappa_{l}(g_{a}^{\left(l\right)},g_{b}^{\left(l\right)}),\kappa_{l}(g_{b}^{\left(l\right)},g_{c}^{\left(l\right)}\right\}$ from both and add *M *to the "direct route" from $g_{a}^{\left(l\right)}$ to $g_{c}^{\left(l\right)}$, *i.e*., set

$$\begin{array}{lll} {\kappa^{\prime }}_{l}(g_{a}^{\left(l\right)},g_{b}^{\left(l\right)}) & \leftarrow & \kappa_{l}(g_{a}^{\left(l\right)},g_{b}^{\left(l\right)})-M\\ {\kappa^{\prime }}_{l}(g_{b}^{\left(l\right)},g_{c}^{\left(l\right)}) & \leftarrow & \kappa_{l}(g_{b}^{\left(l\right)},g_{c}^{\left(l\right)})-M\\ {\kappa^{\prime }}_{l}(g_{a}^{\left(l\right)},g_{c}^{\left(l\right)}) & \leftarrow & \kappa_{l}(g_{a}^{\left(l\right)},g_{c}^{\left(l\right)})+M \end{array}$$

Because *d *is a metric distance, implying

$$d(g_{a}^{\left(l\right)},g_{b}^{\left(l\right)})+d(g_{b}^{\left(l\right)},g_{c}^{\left(l\right)})\geq d(g_{a}^{\left(l\right)},g_{c}^{\left(l\right)})$$

it holds that

$$\begin{array}{ll} & d(g_{a}^{\left(l\right)},g_{b}^{\left(l\right)})\cdot \kappa_{l}(g_{a}^{\left(l\right)},g_{b}^{\left(l\right)})+d(g_{b}^{\left(l\right)},g_{c}^{\left(l\right)})\cdot {\kappa^{\prime }}_{l}(g_{b}^{\left(l\right)},g_{c}^{\left(l\right)})+d(g_{a}^{\left(l\right)},g_{c}^{\left(l\right)})\cdot {\kappa^{\prime }}_{l}(g_{a}^{\left(l\right)},g_{c}^{\left(l\right)})\\ = & d(g_{a}^{\left(l\right)},g_{b}^{\left(l\right)})\cdot {\kappa^{\prime }}_{l}(g_{a}^{\left(l\right)},g_{b}^{\left(l\right)})+d(g_{b}^{\left(l\right)},g_{c}^{\left(l\right)})\cdot \kappa_{l}(g_{b}^{\left(l\right)},g_{c}^{\left(l\right)})+d(g_{a}^{\left(l\right)},g_{c}^{\left(l\right)})\cdot \kappa_{l}(g_{a}^{\left(l\right)},g_{c}^{\left(l\right)})\\ & -M\cdot \left[d(g_{a}^{\left(l\right)},g_{b}^{\left(l\right)})+d(g_{b}^{\left(l\right)},g_{c}^{\left(l\right)})-d(g_{a}^{\left(l\right)},g_{c}^{\left(l\right)})\right]\\ \leq & d(g_{a}^{\left(l\right)},g_{b}^{\left(l\right)})\cdot \kappa_{l}(g_{a}^{\left(l\right)},g_{b}^{\left(l\right)})+d(g_{b}^{\left(l\right)},g_{c}^{\left(l\right)})\cdot \kappa_{l}(g_{b}^{\left(l\right)},g_{c}^{\left(l\right)})+d(g_{a}^{\left(l\right)},g_{c}^{\left(l\right)})\cdot \kappa_{l}(g_{a}^{\left(l\right)},g_{c}^{\left(l\right)}) \end{array}$$

from which it follows that

$$\sum_{u,v}d(g_{u}^{\left(l\right)},g_{v}^{\left(l\right)})\cdot \kappa_{l}(g_{u}^{\left(l\right)},g_{v}^{\left(l\right)})\geq \sum_{u,v}d(g_{u}^{\left(l\right)},g_{v}^{\left(l\right)})\cdot {\kappa^{\prime }}_{l}(g_{u}^{\left(l\right)},g_{v}^{\left(l\right)})$$

Set

$$\begin{array}{lll} \kappa_{l}(g_{a}^{\left(l\right)},g_{b}^{\left(l\right)}) & \leftarrow & {\kappa^{\prime }}_{l}(g_{a}^{\left(l\right)},g_{b}^{\left(l\right)})\\ \kappa_{l}(g_{b}^{\left(l\right)},g_{c}^{\left(l\right)}) & \leftarrow & {\kappa^{\prime }}_{l}(g_{b}^{\left(l\right)},g_{c}^{\left(l\right)})\\ \kappa_{l}(g_{a}^{\left(l\right)},g_{c}^{\left(l\right)}) & \leftarrow & {\kappa^{\prime }}_{l}(g_{a}^{\left(l\right)},g_{c}^{\left(l\right)}) \end{array}$$

If ${\kappa^{\prime }}_{l}(g_{c}^{\left(l\right)},g_{a}^{\left(l\right)})>0$, go to Step 2. Otherwise, repeat Step 3 for another triplet of types fulfilling the condition. If none exists, *STOP*.

At each step, $\sum_{u,v}d(g_{u}^{\left(l\right)},g_{v}^{\left(l\right)})\kappa_{l}(g_{u}^{\left(l\right)},g_{v}^{\left(l\right)})$ decreases or remains constant, yielding

$$\sum_{u,v}d(g_{u}^{\left(l\right)},g_{v}^{\left(l\right)})\cdot \mu_{l}(g_{u}^{\left(l\right)},g_{v}^{\left(l\right)})\geq \sum_{u,v}d(g_{u}^{\left(l\right)},g_{v}^{\left(l\right)})\cdot \kappa_{l}(g_{u}^{\left(l\right)},g_{v}^{\left(l\right)})$$

After completion, either $\sum_{v}\kappa_{l}(g_{u}^{\left(l\right)},g_{v}^{\left(l\right)})=0$ or $\sum_{v}\kappa_{l}(g_{v}^{\left(l\right)},g_{u}^{\left(l\right)})=0$ or both hold for all *u*, meaning that no type is both a source and a sink. The net quasi-shift $\sum_{v}\kappa_{l}(g_{u}^{\left(l\right)},g_{v}^{\left(l\right)})-\sum_{v}\kappa_{l}(g_{v}^{\left(l\right)},g_{u}^{\left(l\right)})$ for each *u *remains constant throughout the algorithm, equaling $P(g_{u}^{\left(l\right)})-Q(g_{u}^{\left(l\right)})$ by Lemma 2. Thus the quasi-shifts *κ*_*l*_($g_{u}^{\left(l\right)}$, $g_{u}^{\left(l\right)}$) fulfill the properties, as claimed.   ■

***Lemma 5***: *The quasi-shifts κ*_*l*_($g_{u}^{\left(l\right)}$, $g_{u}^{\left(l\right)}$) *constructed in Lemma 4 specify a shift transformation s*_*l*_(***P***^(*l*)^, ***Q***^(*l*)^) *for locus l for which it holds that*

$$\sum_{u,v}d(g_{u}^{\left(l\right)},g_{v}^{\left(l\right)})\cdot \mu_{l}(g_{u}^{\left(l\right)},g_{v}^{\left(l\right)})\geq \Delta_{s_{l}}(P^{\left(l\right)},Q^{\left(l\right)})$$

*Proof*: As proven in Lemma 4, for the quasi-shifts $\kappa_{l}(g_{u}^{\left(l\right)},g_{v}^{\left(l\right)})$ it holds that

$$\sum_{v}\kappa_{l}(g_{u}^{\left(l\right)},g_{v}^{\left(l\right)})-\sum_{v}\kappa_{l}(g_{v}^{\left(l\right)},g_{u}^{\left(l\right)})=P(g_{u}^{\left(l\right)})-Q(g_{u}^{\left(l\right)})$$

and either $\sum_{v}\kappa_{l}(g_{u}^{\left(l\right)},g_{v}^{\left(l\right)})=0$ or $\sum_{v}\kappa_{l}(g_{v}^{\left(l\right)},g_{u}^{\left(l\right)})=0$ or both. There are three cases:

$$\begin{array}{ll} \text{If} & \sum_{v}\kappa_{l}(g_{u}^{\left(l\right)},g_{v}^{\left(l\right)})>0\\ \text{then} & \sum_{v}\kappa_{l}(g_{u}^{\left(l\right)},g_{v}^{\left(l\right)})=P(g_{u}^{\left(l\right)})-Q(g_{u}^{\left(l\right)})\\ \text{If} & \sum_{v}\kappa_{l}(g_{v}^{\left(l\right)},g_{u}^{\left(l\right)})>0\\ \text{then} & \sum_{v}\kappa_{l}(g_{v}^{\left(l\right)},g_{u}^{\left(l\right)})=Q(g_{u}^{\left(l\right)})-P(g_{u}^{\left(l\right)})\\ \text{If} & \sum_{v}\kappa_{l}(g_{u}^{\left(l\right)},g_{v}^{\left(l\right)})=\sum_{v}\kappa_{l}(g_{v}^{\left(l\right)},g_{u}^{\left(l\right)})=0\\ \text{then} & P(g_{u}^{\left(l\right)})=Q(g_{u}^{\left(l\right)}) \end{array}$$

These three cases can be combined to the expression

$$\sum_{v}\kappa_{l}(g_{u}^{\left(l\right)},g_{v}^{\left(l\right)})=P(g_{u}^{\left(l\right)})-\min\left\{P(g_{u}^{\left(l\right)}),Q(g_{u}^{\left(l\right)})\right\}$$

Therefore, the quasi-shifts *κ*_*l*_($g_{u}^{\left(l\right)}$, $g_{u}^{\left(l\right)}$) fulfill the definition of alpha shift transformation at locus *l*. Defining the shift $s_{l}(g_{u}^{\left(l\right)},g_{v}^{\left(l\right)}):=\kappa_{l}(g_{u}^{\left(l\right)},g_{v}^{\left(l\right)})$ and denoting $\Delta_{s_{l}}(P^{\left(l\right)},Q^{\left(l\right)}):=\sum_{u,v}d(g_{u}^{\left(l\right)},g_{v}^{\left(l\right)})\cdot s_{l}(g_{u}^{\left(l\right)},g_{v}^{\left(l\right)})$, it follows from Lemma 4 that    ■

$$\sum_{u,v}d(g_{u}^{\left(l\right)},g_{v}^{\left(l\right)})\cdot \mu_{l}(g_{u}^{\left(l\right)},g_{v}^{\left(l\right)})=\Delta_{s_{l}}(P^{\left(l\right)},Q^{\left(l\right)})$$

With the help of the lemmata, Proposition 2 can now be proven:

***Proof of Proposition 2***: Let *s*(***P***, ***Q***) be a shift transformation between the two *L*-locus genotypic structures. Denoting the *L*-locus types as *G*_*x *_or *G*_*y*_, their projections to locus *l *as $G_{x}^{\left(l\right)}$ or $G_{y}^{\left(l\right)}$, and the various single-locus types at locus *l *as $g_{u}^{\left(l\right)}$ or $g_{u}^{\left(l\right)}$, it holds that

$$\begin{array}{lll} \Delta_{s}(P,Q) & = & \sum_{x,y}d(G_{x},G_{y})\cdot s(G_{x},G_{y})\\ & = & \sum_{x,y}\left[\frac{1}{L}\sum_{l=1}^{L}d(G_{x}^{\left(l\right)},G_{y}^{\left(l\right)})\right]\cdot s(G_{x},G_{y})\\ & = & \frac{1}{L}\sum_{l=1}^{L}\sum_{u,v}d(g_{u}^{\left(l\right)},g_{v}^{\left(l\right)})\cdot \mu_{l}(g_{u}^{\left(l\right)},g_{v}^{\left(l\right)})\\ & \leq & \frac{1}{L}\sum_{l=1}^{L}\Delta_{s_{l}}(P^{\left(l\right)},Q^{\left(l\right)})\\ & \leq & \frac{1}{L}\sum_{l=1}^{L}\Delta (P^{\left(l\right)},Q^{\left(l\right)}) \end{array}$$

where *s*_*l*_(***P***^(*l*)^, ***Q***^(*l*)^) is the shift transformation constructed in Lemma 4. Since the inequality holds in particular if *s*(***P***, ***Q***) is a minimal shift transformation, it follows, as claimed, that    ■

$$\Delta (P,Q)\geq \frac{1}{L}\sum_{l=1}^{L}\Delta (P^{\left(l\right)},Q^{\left(l\right)})$$

Equality holds in Proposition 2 whenever the marginal sums for each locus *l *= 1,...,*L *specify a minimal shift transformation, *i.e*., when $\sum_{u,v}d(g_{u}^{\left(l\right)},g_{v}^{\left(l\right)})\cdot \mu_{l}(g_{u}^{\left(l\right)},g_{v}^{\left(l\right)})=\Delta (P^{\left(l\right)},Q^{\left(l\right)}).$.

## Authors' contributions

HRG conceived of the approach and drafted the Background and Methods. EG formulated and proved the Theorem, programmed the software, analyzed the data, and drafted the Results and Appendices. Both authors read and approved the final manuscript.
